# The Immunological and Allergen Profiles of Patients with Atopic Dermatitis or Psoriasis

**DOI:** 10.3390/medicina58030367

**Published:** 2022-03-01

**Authors:** Magdalena Krupka-Olek, Andrzej Bożek, Aleksandra Kawczyk-Krupka

**Affiliations:** 1 Clinical Department of Internal Diseases and Geriatrics, Chair of Internal Diseases, Dermatology and Allergology in Zabrze, Medical University of Silesia, 40-055 Katowice, Poland; magda.krupka94@gmail.com; 2 Department of Internal Medicine, Angiology and Physical Medicine, Centre for Laser Diagnostics and Therapy, Medical University of Silesia, 40-055 Katowice, Poland; akawczyk@gmail.com

**Keywords:** atopic dermatitis, IgE, psoriasis

## Abstract

Background and objectives: Atopic dermatitis (AD) and psoriasis (PS) are systemic inflammatory diseases with complex and distinct immune mechanisms. That the same factors may aggravate both diseases cannot be ruled out. The aim of this study was to assess the potential differences between a sensitization to inhaled allergens and the immunological profiles of patients diagnosed with AD and PS in comparison with healthy controls. Materials and methods: A total of 139 patients with AD, 115 with PS, and 142 controls were included in the prospective study. Patients were eligible if they were diagnosed with mild to severe AD or PS and between 18 and 65 years of age. In all the participants, the serum concentrations of specific IgE (sIgE) for common inhaled allergens were measured. In all the subjects, the cytokine serum blood profiles for TNF-α, IFN-γ, Il-2, Il-4, Il-5, Il-6, Il-8, Il-12, Il-17, Il-18, Il-22, and Il-24 were measured via an ELISA. Results: The patients with AD had positive sIgE results more frequently than the patients with PS and the controls (113 vs. 36 vs. 21, respectively). A sensitization to mites was dominant in the patients with AD (*p* < 0.05), and a sensitization to *Aspergillus* was dominant in the patients with PS (*p* < 0.05). The patients with multiple allergies to inhaled allergens had a lower risk of developing PS (OR = 0.65; 95% CI: 0.43–0.86) but a greater risk of severe AD (OR = 3.77; 95% CI: 3.25–3.96). The mean concentrations of the most tested cytokines were comparable in the patients with AD and PS. However, high serum concentrations of Il-4, Il-5, and Il-6 were only dominant in the AD group. There were no relationships between the increased serum concentrations of individual cytokines and allergies to the individually examined allergens. Conclusion: Inhalation-dependent IgE sensitizations were prevalent in the AD patients but were also possible in the PS patients; they were often without clinical manifestations in the latter group. The investigated cytokine profiles indicated their high convergence in the studied patients and confirmed the active inflammatory nature of AD and PS.

## 1. Introduction

The widespread prevalence of allergies, currently estimated to be one third of the population, indicates the need for detailed research to understand the pathogenesis of allergic diseases such as atopic dermatitis (AD) [1,2]. AD is heterogeneous; its pathogenesis is complex and not defined in detail [1,2]. AD is an early childhood disease and remains in adulthood in several patients. Briefly, the disease is driven by Th2 cells with an overproduction of Il-4, Il-13, and IgE. However, in a few forms, low IgE values and a high Th1 activity have been observed [1,3].

It is extremely important to note that the commonly recognized significance of allergens in inducing or exacerbating a disease has a different role in AD. Often, environmental allergens are overlooked when focusing on food allergens. There are many data indicating their important role, often as exacerbating factors in allergic skin diseases. For example, mite allergens play a special role due to their widespread distribution; frequent allergies to *D. pteronyssinus* and *D. farinae* have been reported [4,5]. There are still relatively few data on the incidence of this allergy in other skin diseases and its potential impact on the course of these diseases. Despite comprehensive information on the mechanisms of other skin diseases such as psoriasis, the ultimate presence of allergies and their impacts on such skin diseases have not yet been determined. It is believed that psoriasis is a disease that is not associated with atopic dermatitis, as evidenced by their extremely rare coexistence [6,7]. However, it does not exclude the coexistence of, for example, an allergy to environmental, food, or contact allergens, which is a factor in individual works [8,9,10].

The aim of the study was to assess the potential differences between a sensitization to inhaled allergens and the immunological profiles of patients diagnosed with chronic atopic dermatitis, patients diagnosed with psoriasis, and healthy volunteers. The possible impact of an allergy on a specific allergen and of the immunological profile on the severity of the analyzed skin diseases were assessed.

## 2. Materials and Methods

### 2.1. Patients

In this prospective study, patients were included if they were diagnosed with AD or PS, were between 18 and 65 years of age, and had a mild to severe form of AD, according to the objective SCORing Atopic Dermatitis (SCORAD) index or mild to severe PS, according to the objective Psoriasis Area Severity Index (PASI). The patients were observed for 12 months or longer. The study was performed at the Dermatology and Allergology Outpatient Clinic in Zabrze, the Medical University of Silesia. All the participants provided written consent to participate in the study.

A diagnosis of AD was based on the clinical characteristics of the patient according to the criteria of Hanifin and Rajka [11]. PS was confirmed based on a typical morphology for psoriasis vulgaris and the history of the patient [12,13].

Additionally, healthy volunteers without skin diseases, who were consistent with the other groups in terms of sex and age, were included in the study as a control group.

The exclusion criteria were as follows: ambiguous skin changes; failure to meet all the diagnostic criteria for a given disease; other skin diseases; other serious diseases that may have affected the results; and a lack of patient consent. The characteristics of the patients are presented in Table 1.

### 2.2. Methods

All the patients and controls underwent a thorough dermatological examination with the possible determination of the disease stage using the SCORAD (SCORing Atopic Dermatitis) scale for the AD patients and the PASI (Psoriasis Area Severity Index) scale for the PS patients. In all subjects, the serum blood profiles were evaluated for allergen-specific IgE for the following inhaled allergens: *D. pteronyssinus*; *D. farinae*; timothy grass; rye; alder; birch; hazel; ambrosia; mugwort; cats; dogs; *Alternaria alternata*; *Cladosporium herbarum*; *Aspergillus fumigatus*; and *Penicillium notatum.* The determinations were achieved using the immunoenzymatic method using a Thermo Fisher system. A concentration of > 0.35 IU/L was considered to be positive according to the manufacturer’s recommendation. In all the subjects, the cytokine serum blood profiles for TNF-α, IFN-γ, Il-2, Il-4, Il-5, Il-6, Il-8, Il-12, Il-17, Il-18, Il-22, and Il-24 were measured via an ELISA (Thermo Fisher Scientific, Waltham, MA, USA). The study was approved by the local bioethics committee of the Medical University of Silesia in Poland (PCN/0022/KB1/24/21).

#### Statistical Analysis

A statistical analysis was performed using Statistica 8.2 (SaftPOl, Krakow, Poland). A Wilcoxon test or a Student’s *t*-test was performed to compare the relevant variables. The correlations between the analyzed parameters were assessed using a Spearman’s test. The odds ratio (OR) and 95% confidence interval (CI) were estimated for the variables. A *p*-value < 0.05 was considered to indicate a significance.

## 3. Results

In total, we evaluated 139 patients with atopic dermatitis (AD), 115 patients with psoriasis (PS), and 142 healthy controls. All the patients were Caucasian. The obtained results demonstrated that patients with AD significantly more frequently had a positive inhaled allergy based on positive sIgE results compared with patients with PS and the control volunteers. The detailed data are presented in Table 2. In patients with PS, positive results for the tested inhaled allergens occurred much less frequently and the profile was partially different from those of the patients with AD and the control volunteers.

The severity of AD based on the results of the SCORAD scale correlated with the number of sensitizations to the tested allergens (*p* < 0.05). The patients with allergies to house dust mites and the patients with a presence of multiple allergies (more than three positive allergens) had a greater risk of severe AD (SCORAD > 50 points) (OR = 2.87; 95% CI: 2.12–3.06 and OR = 3. 77; 95% CI: 3.25–3.96, respectively).

The severity of PS based on the results of the PASI scale did not correlate with the presence of a sensitization to the examined allergens or their number (*p* = 0.23 or *p* = 0.16). However, patients with multiple allergies had a lower risk of developing PS (OR = 0.65; 95% CI: 0.43–0.86). The persistence of mugwort or *Aspergillus fumigatus* allergies necessarily increased the risk of developing AD and PS (OR = 1.54; 95% CI: 1.44–1.65 and OR = 1.74; 95% CI: 1.38–1.85, respectively). The other allergens did not have a direct impact on the incidence of PS or AD or their severity.

The lack of allergies to the examined allergens did not affect the appearance of PS but did reduce the risk of AD (OR = 0.49; 95% CI: 0.38–0.68).

### Immunological Profile

The analysis of the concentrations of the tested cytokines in the blood serum showed significant differences among the studied groups. The results are presented in Table 3.

Most of the mean concentrations of the tested cytokines were comparable in the patients with AD and PS and significantly higher than those in the control group. However, high serum concentrations of Il-4, Il-5, and Il-6 were only dominant in the AD group.

An additional subgroup analysis of extrinsic and intrinsic AD and PS indicated additional differences in the concentration of Il-2, Il-4, Il-5, and Il-17 between the AD endotypes. The results are presented in Table 4.

The correlation analysis did not show any relationship between the increased serum concentrations of the individual cytokines and allergies to the individual allergens in patients with AD or PS. However, in patients with AD, a simultaneous increase in the serum concentrations of Il-4 and Il-6 correlated with the presence of a mite or grass allergy with coefficients of 0.82 and 0.79, respectively, in the Spearman’s rank correlation test at *p* < 0.05.

There were no differences between the concentrations of the analyzed cytokines in the group of sensitized and non-sensitized psoriatic patients.

## 4. Discussion

The present study confirmed a significant number of AD patients with allergies to the tested inhaled allergens, as well as the occurrence of such allergies to a lesser extent in patients with PS. However, as in the control group, in most cases of PS there were no clinical symptoms of allergies and the analyzed positive serum concentration of sIgE was relatively low. Currently, there are few data on the prevalence of allergies in patients with PS. This is the first study to compare inhalation allergy assessments and cytokine profiles in such a large group of AD or PS patients and controls. Previous studies have mainly focused either on patients with psoriasis only or on the assessment of individual allergens [10,14]. Only Barilo et al. assessed the incidence of allergies in AD or PS and showed, similar to our study, a greater incidence of allergies in patients with AD [15]. However, there was no assessment of the cytokine levels.

The comparative frequency of allergies to mugwort and *Aspergillus fumigatus* in both the studied groups is interesting. The patients did not associate the disease severity with an exposure to these allergens in either the AD or PS groups (data not published). An exposure to environmental allergens appears to be able to influence AD but this relationship is not obvious in PS [1,2,4]. There are a few examples in the literature regarding the influence of mite allergens on the expression of atopic dermatitis, but there are no such data in relation to other inhaled allergens or psoriasis [4,16]. Kanemaru et al. admitted that house dust mites are a major allergen that causes allergic diseases such as AD, but the regulatory mechanisms of mite-induced immune responses are incompletely understood. In an animal model, it was suggested that the C-type lectin receptor Clec10a in mice and the similar Asgr1 protein in humans, regulate the inflammation associated with mite-induced dermatitis [17]. In our study, no effect of a dust allergy on the severe course of AD was observed.

The similarity of the serum cytokine concentration profiles confirmed the inflammatory nature of both diseases. The higher concentrations of Il-4, Il-5, and Il-6 in AD could emphasize its dependent nature, which has been repeatedly confirmed in the literature [1,2,18]. AD is associated with T_H_2-polarized CD4^+^ T cells that can overproduce IL-6, IL-5, IL-4, and IL-13. IL-6 activates IL-4-producing CD4^+^ T cells, induces the acute-phase response as well as T and B cell proliferation, and is involved in the development of T_H_17 cells [1,18,19].

The hypothesis regarding the important role of T-17 lymphocytes as a common link for PS and AD has not yet been clearly confirmed in the obtained results [20,21]. In the present study, the concentration of Il-17 was particularly represented in the patients with PS and less represented in the patients with AD (but without statistical significance). This may be because T helper 17 cells produce cytokines, which affect keratinocyte proliferation, stimulate inflammation, and play a key role in PS [20].

The observed lower mean concentrations of Il-4 and Il-5 as well as higher Il-17 in intrinsic AD compared with the extrinsic form may suggest a similarity in the molecular and immunological levels between this non-allergic form of AD and PS. Our group of patients was too small to allow for a final assessment of this phenomenon.

There were a few limitations of the study, as is usual in such projects, such as the groups too small to analyze, not allowing for a precise epidemiological assessment in terms of the assessment of the tested allergies and producing only pilot results. Allergen-specific IgE to food allergies were omitted because such allergies are not of decisive importance in adults [19]. However, such data may be interesting. The limited profile of the tested cytokines could determine the final results, especially because they were determined at one time point without tracking their dynamics. A study of the relationship between the severity of the disease and changes in the cytokine concentrations would be interesting and will be the subject of further research by the authors.

## 5. Conclusions

Inhalation-dependent IgE sensitizations were prevalent in AD patients, but they also occurred in PS patients, often without clinical manifestations in the latter group. The investigated cytokine profiles indicated their high convergence in the studied patients and confirmed the active inflammatory nature of the analyzed diseases. Further research is needed to clarify a possible link to allergies in psoriasis, especially in the clinical picture of the disease.

## Figures and Tables

**Table 1:** Characteristics of the study patients.

	Patients with AD *n* = 139	Patients with PS *n* = 115	Healthy Volunteers *n* = 142
Age	31.5 ± 5.4	42.7 ± 59.1	38.7 ± 5.4
Women (%)	54 (39)	50 (44)	59 (42)
Mean time of disease duration (Yrs.)	11.3	8.8	-
Smokers	14 (10)	19 (17)	21 (15)
Rural place of living	31 (22)	21 (18)	32 (23)
Atopic diseases in medical history	121 (87)	26 (23)	29 (20)
Atopy in the family	59 (43)	21 (18)	42 (30)
BMI > 25	14 (10)	35 (31)	24 (17)
Asthma	45 (32)	14 (12)	9 (6)
Allergic rhinitis	58 (42)	23 (20)	20 (14)
Additional AD	-	1	-
Additional PS	1 (1)	-	-
Other additional dermatoses:	24 (17)	23 (20)	-
*Onychomycosis*	9 (6)	10 (9)	
Nevi pigmentosi *	11 (8)	12 (10)	
*Vitiligo*	1 (1)	0	-
*Rosacea*	3 (2)	1 (1)	-
Mean SCORAD ± SD	48 ± 16	-	
Mean PASI ± SD	-	23 ± 9	
Anaphylactic shock in the patient history	7 (5)	1 (1)	1 (1)
Systemic therapy	97 (70)	67 (58)	-
Corticosteroids	54 (39)	0	-
Antihistamines	95 (68)	6 (5)	-
Biological drugs	8 (6)	32 (28)	-
Cyclosporin	34 (24)	16 (14)	-
Puva	28 (20)	18 (16)	-
Methotrexate	3 (2)	11 (10)	-
Others	1 (1)	3 (3)	-

AD: atopic dermatitis; PS: psoriasis; BMI: body mass index; * melanoma excluded.

**Table 2:** Positive results of allergen-specific IgE for inhaled allergens in the studied groups.

	Number of Positive Results *		
Allergen	Patients with AD *n* = 139	Patients with PS *n* = 115	Healthy Volunteers *n* = 142
*D. pteronyssinus*	67 ^	9	11
*D. farinae*	56 ^	11	13
Timothy grass	42 ^	4	9
Rye	38 ^	2	12
Alder	33 ^	9	8
Birch	45 ^	8	6
Hazel	32 ^	3	7
Ambrosia	14 ^	1	2
Mugwort	13	10	2 ^^
Cats	37 ^	4	7
Dogs	23 ^	5	4
*Alternaria alternata*	34 ^	5	8
*Cladosporium herbarum*	29 ^	6	5
*Aspergillus fumigatus*	11	12	3 ^^
*Penicillium notatum*	14	4	2
Multiple allergies	98	9	2
Any positive sIgE	113 ^	36	21

* sIgE > 0.35 kU/L; ^ more frequent positive results of specific IgE in patients with AD vs. patients with PS and vs. the controls (*p* < 0.01); ^^ less frequent positive results of specific IgE in the controls than in patients with AD and PS (*p* < 0.05) and comparable results between patients with AD and PS (*p* > 0.05).

**Table 3:** The cytokine profiles of the blood serum of the studied patients and controls.

Cytokines	Patients with AD *n* = 139	Patients with PS *n* = 115	Healthy Volunteers *n* = 142
	Values Are the Mean ± SD		
TNF-α (pg/mL)	30.7 ± 9.5	36.9 ± 15.1	8.7 ± 5.5 *
IFN-γ (IU/mL)	0.65 ± 0.32	0.71 ± 0.43	0.51 ± 0.98
Il-2 (pg/mL)	258.4 ± 25.4	150.4 ± 87.2	65 ± 29.5 *
Il-4 (pg/mL)	184.5 ± 92 **	90.4 ± 36.7	67.5 ± 30.1
Il-5 (pg/mL)	984 ± 450 **	205 ± 450	80.4 ± 19
Il-6 (pg/mL)	78.1 ± 19.3 **	36.3 ± 24.5	28.5 ± 14.5
Il-8 (pg/mL)	18.3 ± 10.1	27.5 ± 11.2	12.1 ± 5.6
Il-12 (pg/mL)	38.2 ± 16.2	50.3 ± 14.2	16.1 ± 8.6 *
Il-17 (pg/mL)	9.5 ± 3.2	14.1 ± 5.2	2.1 ± 0.9 *
Il-18 (pg/mL)	182.1 ± 67.5	133.3 ± 70.4	29.4 ± 9.3 *
Il-22 (pg/mL)	23.2 ± 4.4	47.3 ± 11.1	2.5 ± 0.9 *
Il-24 (pg/mL)	120.3 ± 80.5	187 ± 73.1	18.2 ± 5.9 *

* Significantly lower mean serum concentration in the control group vs. the other groups (*p* < 0.05); ** significantly higher mean serum concentration in the AD group vs. the PS and control groups (*p* < 0.05).

**Table 4:** The cytokine profiles of the blood serum of the patients with extrinsic AD, intrinsic AD, and PS.

Cytokines	Patients with Extrinsic AD *n* = 128	Patients with Intrinsic AD *n* = 11	Patients with PS *n* = 115
	Values Are the Mean ± SD		
TNF-α (pg/mL)	29.1 ± 10.2	34.2 ± 6.5	36.9 ± 15.1
IFN-γ (IU/mL)	0.61 ± 0.42	0.74 ± 0.21	0.71 ± 0.43
Il-2 (pg/mL)	274.9 ± 58.4 *	172.3 ± 34.2	150.4 ± 87.2
Il-4 (pg/mL)	198.2 ± 89	175.4 *± 56.3*	90.4 *± 36.7 ^*
Il-5 (pg/mL)	902 ± 386 *	290 ± 312	205 ± 450
Il-6 (pg/mL)	80.2 ± 20.8	69.7 ± 23.1	36.3 ± 24.5 ^
Il-8 (pg/mL)	16.9 ± 9.4 ^^	23.1 ± 6.4	27.5 ± 11.2
Il-12 (pg/mL)	33.9 ± 15.3	38.9 ± 9.1	50.3 ± 14.2 *
Il-17 (pg/mL)	8.4 ± 4.1 ^^	12.9 ± 6.2	14.1 ± 5.2
Il-18 (pg/mL)	195 ± 88.4	187 ± 85.2	133.3 ± 70.4 ^
Il-22 (pg/mL)	21.2 ± 6.5	23.1 ± 11.4	47.3 ± 11.1 **
Il-24 (pg/mL)	119.8 ± 90.2	132.5 ± 90.5	187 ± 73.1 **

* Significantly higher mean serum concentration in the extrinsic AD group vs. the intrinsic AD and PS groups (*p* < 0.05); ^ significantly lower mean serum concentration in the PS group vs. the other groups (*p* < 0.05); ^^ significantly lower mean serum concentration in the extrinsic AD group vs. the other groups (*p* < 0.05); ** significantly higher mean serum concentration in the PS group vs. the other groups.

## Data Availability

The data presented in this study are available on request from the corresponding author. The data are not publicly available due to ethical restrictions.
